# Identification of *ZmSNAC06*, a Maize NAC Family Transcription Factor with Multiple Transcripts Conferring Drought Tolerance in *Arabidopsis*

**DOI:** 10.3390/plants14010012

**Published:** 2024-12-24

**Authors:** Fei Wang, Yong Chen, Ruisi Yang, Ping Luo, Houwen Wang, Runze Zhang, Wenzhe Li, Ke Yang, Xinlong Xu, Zhuanfang Hao, Xinhai Li

**Affiliations:** 1 State Key Laboratory of Crop Gene Resources and Breeding, Institute of Crop Sciences, Chinese Academy of Agricultural Sciences, Beijing 100081, China; wangfei19990908@163.com (F.W.); yangruisixj@163.com (R.Y.); luoping987@126.com (P.L.); whw15797929108@163.com (H.W.); zrz04222022@163.com (R.Z.); liwz16603632000@163.com (W.L.); yk864078904@163.com (K.Y.); xuxinlong0112@163.com (X.X.); 2 College of Life Science, South China Agricultural University, Guangzhou 510642, China; chenyong9576@126.com

**Keywords:** maize (*Zea mays* L.), *ZmSNAC06*, transcription factor, drought tolerance

## Abstract

Drought is one of the most serious environmental stresses affecting crop production. NAC transcription factors play a crucial role in responding to various abiotic stresses in plants. Here, we identified a maize NAC transcription factor, *ZmSNAC06*, between drought-tolerant and drought-sensitive inbred lines through RNA-seq analysis and characterized its function in *Arabidopsis*. *ZmSNAC06* had five transcripts, of which *ZmSNAC06*-T02 had a typical NAC domain, while ZmSNAC06-P02 was localized in the nucleus of maize protoplasts and had transactivation activity in yeasts. The expression of *ZmSNAC06* in maize was induced by drought. The overexpression of *ZmSNAC06*-T02 in *Arabidopsis* resulted in hypersensitivity to abscisic acid (ABA) at the germination stage, and overexpression lines exhibited higher survival rates and higher antioxidant enzyme activities compared with the wild-type under drought stress. These results suggest that *ZmSNAC06* acts as a positive regulator in drought tolerance and may be used to improve drought tolerance in crops.

## 1. Introduction

Global climate change has caused a lot of uncertainty in crop growth; permanent or sudden drought often occurs in the main production areas of grain crops, which seriously decreases the agricultural output worldwide [1]. In response to drought stress, plants have developed a variety of adaptive strategies, including drought escape, drought avoidance and drought tolerance [2]. Drought tolerance is a complex trait that involves different tissues and metabolic pathways. In the regulatory network of the plant response to stress, transcription factors (TFs) activate or inhibit the expression of stress-related genes by binding to cis-acting promoter elements [3].

NAC (*NAM*, *ATAF1/2* and *CUC2*) is a plant-specific transcription factor (TF) family. Members of the NAC family have a highly conserved N-terminal NAC binding domain, which contains approximately 160 amino acids [4,5]. The NAC binding domain can be divided into five subdomains (A-E) [6]. Subdomain C belongs to DNA-binding regions [7], subdomain D belongs to the nuclear localization signal region and subdomain A belongs to the oligomerization site region [8]. Subdomains B and E are relatively variable and may contribute to the function diversity of the NAC protein [9]. The highly variable C-terminal of the NAC protein is a transcriptional activation/repression region [10]. NAC TFs are involved in the regulation of plant growth and stress responses, and they are widely distributed in various plant genomes. Up to now, a large number of NAC genes have been identified in *Arabidopsis*, rice, tobacco, soybean, wheat and maize genomes [11,12,13,14,15,16].

Some members of the NAC family respond to drought stress based on different molecular mechanisms and have been identified and studied in *Arabidopsis*, rice, soybeans, wheat and maize. As a model plant, *Arabidopsis* has been extensively studied. *NAC016* was negatively involved in the drought stress response, and it inhibited ABA response element binding protein 1 (AREB1) to regulate the drought response in plants [17]. The overexpression of *SNAC3*, *OsNAC5* and *ONAC066* in rice improved drought tolerance and up-regulated the expression of stress-related genes [18,19,20]. *OsNAC2* was identified as combining the promoters of *OsLEA3* (encoding a group 3 late embryogenesis abundant protein) and *OsNCED3* (encoding rate-limiting enzymes in ABA biosynthesis) and positively regulated drought tolerance through an ABA-dependent signaling pathway in rice [21]. *OsNAC016* was phosphorylated by GSK3/SHAGGY-LIKE KINASE2 (GSK2) and OSMOTIC STRESS/ABA-ACTIVATED PROTEIN KINASE8 (SAPK8) to negatively regulate ABA-mediated drought tolerance pathways [22]. *SNAC1* activated the antioxidant system in rice and improved drought tolerance [23]. Compared with the wild-type soybean, the *GmNAC12* overexpression lines showed enhanced tolerance to drought, while *GmNAC12* knockout lines were sensitive to drought [24]. In wheat, *TaSNAC8-6A* with an ABRE motif (TACGTG) in the promoter could interact with TaABFs to enhance drought tolerance [25]; *TaNAC071-A* significantly enhanced drought tolerance by increasing the water use efficiency [26]. There are a large number of NAC genes in the maize genome, but there are few reports on the mechanism of drought tolerance. *ZmNAC55*, *ZmNAC33* and *ZmSNAC13* were identified to improve drought tolerance in transgenic *Arabidopsis* [27,28,29]; *ZmNAC49* inhibited the expression of *ZmMUTE* (a bHLH transcription factor) to reduce the stomatal density and improved drought tolerance in maize [30]. The insertion of miniature inverted repeat transposable elements (MITEs) in the promoter region of *ZmNAC111* was associated with drought tolerance in maize. The overexpression of *ZmNAC111* enhanced drought tolerance in transgenic *Arabidopsis* and maize seedlings [31]. *ZmNAC20* enhanced the drought tolerance of maize by promoting stomatal closure and activating the expression of stress-related genes [32].

The total production of maize in 2020 reached 1.163 billion tons, and it has become the crop with the highest production in the world [33]. Maize production requires sufficient water, especially in the flowering stage, but the availability of fresh water is predicted to decline by 50% in 2050 [34,35]. Thus, there is an urgent demand for improving maize drought tolerance. In this study, a transcription factor, *ZmSNAC06*, was identified through RNA-seq analysis between drought-tolerant (Tie7922 and X178) and drought-sensitive (Ji81162 and CA339) inbred lines. *ZmSNAC06*-T02 played an important regulatory role in the drought stress response, which provided information for the further study of its molecular mechanism.

## 2. Results

### 2.1. Transcriptome Analysis and Identification of ZmSNAC06 in Maize

**Figure 1:**
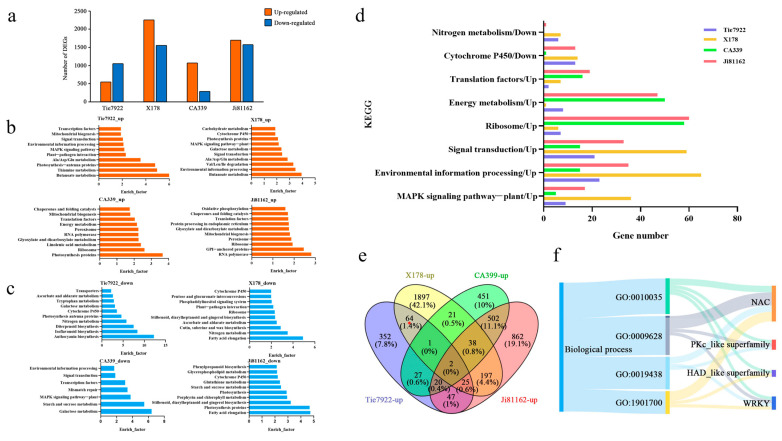
Transcriptome analysis of four inbred maize lines. (**a**) The number of up-regulated or down-regulated genes in four inbred lines under drought stress. (**b**) KEGG map of the up-regulated genes among four inbred lines under drought stress. (**c**) KEGG map of the down-regulated genes among four inbred lines under drought stress. (**d**) The gene expression number of the drought response pathway in four inbred lines. (**e**) Venn diagram of up-regulated genes of four inbred lines under drought stress. “*” means that these 64 genes are upregulated in both Tie7922 and X178. (**f**) GO annotation and family analysis based on the 64 up-regulated genes in Tie7922 and X178.

In this study, the drought-tolerant inbred lines Tie7922 (heterotic group: BSSS group) and X178 (PB group) and drought-sensitive inbred lines CA339 (LRC group) and Ji81162 (BSSS group) were subjected to RNA-seq analysis [36]. We compared the transcriptome data of four inbred lines under well-watered and drought-stressed conditions. It was found that under drought stress, 538 genes were identified as up-regulated and 1044 genes were down-regulated in Tie7922, and there were 2245 genes up-regulated and 1545 genes down-regulated in X178, while there were 1062 genes up-regulated and 280 genes down-regulated in CA339 and 1693 up-regulated genes and 1568 down-regulated genes in Ji81162 (Figure 1a).

We analyzed the pathways for the co-enrichment of drought-tolerant or drought-sensitive materials and found that the number of genes expressed differed based on the drought response pathways in the four inbred lines (Figure 1d). KEGG enrichment analysis showed that up-regulated genes in drought-tolerant materials were mainly enriched in signal transduction and stress metabolite anabolic pathways, while in drought-sensitive materials, up-regulated genes were mainly enriched in translation and photosynthetic/respiratory energy metabolic pathways (Figure 1b). The down-regulated genes of the four materials were also analyzed under drought stress. It was found that some secondary metabolite pathways were prevalent in the four materials, such as the MAPK signaling pathway, environmental information processing and signal transduction, but the drought-tolerant materials Tie7922 and X178 were enriched in nitrogen metabolism pathways, which play an important role in the transition from vegetative growth to reproductive growth (Figure 1c).

Sixty-four genes were identified as up-regulated in both the drought-tolerant materials Tie7922 and X178. Among them, four genes belonged to the NAC family, three genes belonged to the kinase family, two genes belonged to the phosphatase family and one gene belonged to the WRKY family (Figure 1e,f). In the four NAC genes, three of them, including GRMZM2G347043 (*ZmSNAC02*, *ZmSNAC1*, *ZmNAC49*), GRMZM2G014653 (*ZmSNAC04*, *ZmNAC33*, *NAC109*) and GRMZM2G068973 (*ZmSNAC13*, *ZmNAC080308*), have been reported to improve drought tolerance in transgenic *Arabidopsis* or maize [28,29,37,38,39]. Therefore, another NAC gene, which had not yet been characterized, was named as *ZmSNAC06* (GRMZM2G123667) to be studied in this paper.

### 2.2. Bioinformatics and Sequence Characterization of ZmSNAC06

**Figure 2:**
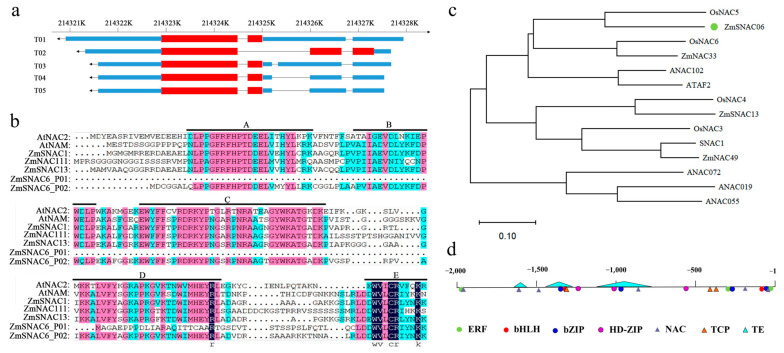
Gene structure, NAC domain sequence alignment, phylogeny and promoter region analysis in *ZmSNAC06*. (**a**) Structure of the *ZmSNAC06* gene transcripts; the blue part represents 5′UTR and 3′UTR, the red part represents the exon of the gene and the black line represents the intron of the gene. (**b**) NAC domain sequence alignment of *ZmSNAC06* and NAC members from other plant species. Identical amino acids are shaded in dark blue, and similar amino acids are shaded in pink or light blue. The locations of the five highly conserved amino acid motifs (A–E) are indicated by black lines. (**c**) Phylogenetic relationships between *ZmSNAC06* and typical stress-responsive NAC proteins. The multiple sequence alignment was performed using ClustalW, and the phylogenetic tree was constructed with MEGA11.0 using the neighbor-joining method. (**d**) Distribution of the transposable elements and binding sites of transcription factors related to stress in the promoter region (~2.0 kb).

Five transcripts were found in *ZmSNAC06* from the Zm-B73-REFERENCE-NAM-5.0 version of the sequence annotation. Differences in the five transcripts resulted in two subtypes of proteins, ZmSNAC06-P01 and ZmSNAC06-P02 (Figure 2a,b). The ZmSNAC06-P02 subtype protein was translated from the *ZmSNAC06*-T02 transcript, which had an open reading frame length of 1080 bp with 359 amino acids, and it had a typical NAC domain, while the ZmSNAC06-P01 subtype protein was translated from four transcripts of *ZmSNAC06*-T01/3/4/5 with an open reading frame length of 750 bp, with 250 amino acids. The ZmSNAC06-P01 subtype protein had only the E subdomain of the NAC domain. Subsequently, *ZmSNAC06*-T02 sequences were cloned from the maize inbred lines Tie7922 and Ji81162. A lot of variations were then identified in the *ZmSNAC06*-T02 gene region between the two lines, including insertion, deletion and single nucleotide polymorphisms. Three amino acid differences were identified in the coding region. Compared with Tie 7922, the 173rd amino acid changed from an aspartic acid into an asparagine, and there were the deletions of the 292nd and 293rd alanines, 317th valine and 318th aspartic acid in Ji81162 (Figure S1). The promoter sequence (~2000 bp upstream of the transcription start site) of the *ZmSNAC06*-T02 transcript between Tie7922 and Ji81162 was completely consistent.

Phylogenetic analysis revealed that *ZmSNAC06*-T02 was more closely related to *OsNAC5*, a known drought tolerance gene (Figure 2c). As a member of the NAC transcription factor family, the promoter sequence in *ZmSNAC06*-T02 displayed three transposable elements and a large number of binding sites of transcription factors related to stress responses and plant growth (Figure 2d). We found that there were six NAC transcription factor recognition motifs in the promoter region, and many recognition motifs of other transcription factors, including bZIP, bHLH and HD-ZIP, were also identified in transposable elements. These elements were perhaps related to the functional regulation of *ZmSNAC06* in maize.

### 2.3. ZmSNAC06 Expression Was Induced by Drought Stress

**Figure 3:**
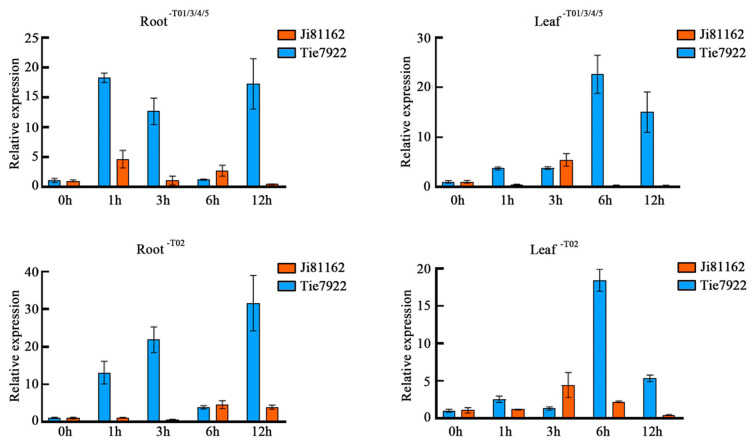
Drought response of *ZmSNAC06* with multiple transcripts in different inbred lines.

Tie7922 and Ji81162, which have a close relationship but are significantly different in terms of drought tolerance, were selected as drought-tolerant and drought-sensitive lines to investigate the stress response of *ZmSNAC06*. qRT-PCR results showed that *ZmSNAC06* was differently expressed in the response pattern of tolerant/sensitive materials. In Tie7922, the expression levels of *ZmSNAC06*-T01/3/4/5 peaked at 1 h and 6 h in the roots and leaves, respectively, and peaks appeared at 1 h and 3 h in the roots and leaves, respectively, in Ji81162. The expression of *ZmSNAC06*-T02 in Tie7922 achieved a maximum level at 12 h and 6 h in the roots and leaves, and it peaked at 6 h and 3 h in the roots and leaves in Ji81162, respectively. The different transcripts of *ZmSNAC06* were up-regulated after PEG treatment at 1 h, 3 h and 12 h in the roots and at 1 h, 6 h and 12 h in the leaves, but the expression in Ji81162 was far less obvious than that in Tie7922 (Figure 3).

### 2.4. ZmSNAC06 Was Localized in the Nucleus and Had Transactivation Activity

**Figure 4:**
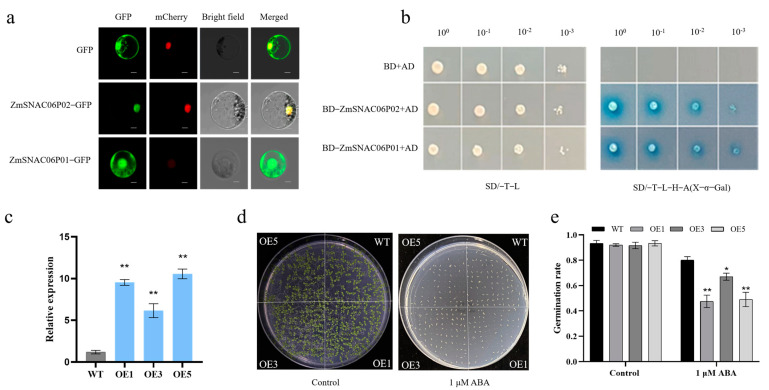
Analysis of the subcellular localization and transactivation activity of ZmSNAC06 and its expression level and germination rate in transgenic *Arabidopsis*. (**a**) Subcellular localization of ZmSNAC06 in maize protoplasts. GFP: Green fluorescent protein; mCherry: nuclear markers; scale bar = 10 μm. (**b**) Transactivation activity of ZmSNAC06 in yeasts. (**c**) qRT-PCR analysis of *ZmSNAC06*-T02 transcript levels in three independent lines. (**d**) Germination phenotype of *Arabidopsis* under normal conditions and ABA treatment. (**e**) Germination rate statistics of *Arabidopsis* under normal conditions and ABA treatment. Significant differences were determined by *t*-test. * *p* < 0.05, ** *p* < 0.01.

Transcription factors are usually located in the nucleus, where they perform DNA-binding and transcriptional activation functions. To determine the subcellular localization of different transcripts in ZmSNAC06, PAN580-GFP and ZmSNAC06-P01/P02-GFP were transformed into maize protoplasts, and it was found that ZmSNAC06-P01 was localized in the cell membrane, nucleus and cytoplasm, whereas, ZmSNAC06-P02 was localized only in the nucleus, indicating that the NAC domain of the ZmSNAC06 protein might affect the subcellular localization (Figure 4a).

Moreover, to study whether the ZmSNAC06-P01/P02 protein had transactivation activity, ZmSNAC06-P01/P02 was fused to the GAL4 DNA-binding domain in the pGBKT7 vector and co-transferred to yeasts with the pGADT7-T vector. The transformants grew normally and turned blue on an SD medium (SD/-Trp-Leu-His-Ade) with X-α-Gal. Both ZmSNAC06-P01/P02 proteins had transactivation activity, indicating that different NAC domains in different transcripts did not affect the transactivation activity of ZmSNAC06 (Figure 4b). The C-terminal of NAC proteins with transcriptional regulatory domains might function to bind to other proteins and thus have transactivation activity.

### 2.5. Germination Sensitivity of Transgenic Arabidopsis to ABA

Since multiple transcripts of *ZmSNAC06* encode two subtypes of P01 or P02 proteins with an identical C-terminal and the difference in the N-terminal is the presence or absence of NAC domains, the structure of the P02 subtype protein basically contains P01. We selected the major model T02 transcript of *ZmSNAC06* for transformation in *Arabidopsis*. Three independent overexpression lines (OE1, OE3 and OE5) were selected based on expression levels for further study (Figure 4c).

To investigate the sensitivity of transgenic plants to ABA, the germination rate was calculated. Under normal conditions, no significant difference in the germination rate was observed between the wild-type (WT) and overexpression lines (Figure 4d). Under a 1 μM ABA treatment, the germination rates of overexpression lines ranged from 48% to 67%, while the germination rate of the WT was 78% (Figure 4e). The overexpression of *ZmSNAC06*-T02 led to the hypersensitivity of transgenic *Arabidopsis* to ABA.

### 2.6. ZmSNAC06-T02 Confers Drought Tolerance in Transgenic Arabidopsis

**Figure 5:**
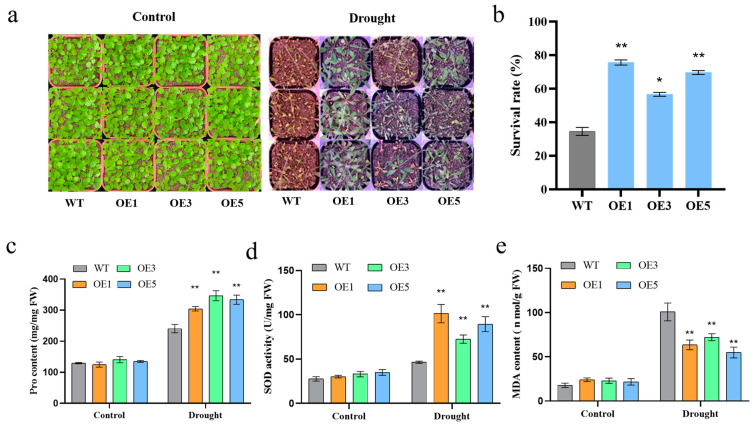
Drought tolerance and physiological indices of transgenic *Arabidopsis* (Pro, SOD, MDA). (**a**) Phenotypes of *ZmSNAC06* overexpression lines and WT. (**b**) Survival rates of *Arabidopsis* after drought treatment. (**c**–**e**) Pro content, SOD activity and MDA content in WT and three overexpression lines were measured before and after 10 days of drought treatment. Significant differences were determined by *t*-test. * *p* < 0.05, ** *p* < 0.01.

To assess the drought tolerance of transgenic *Arabidopsis*, the survival rates of the WT and the three overexpression lines after drought stress were calculated. Overexpressed plants showed stronger growth recovery (Figure 5a). We found that 56% to 77% of transgenic *Arabidopsis* plants recovered from drought stress after rewatering, which was significantly higher than that of the WT, with a survival rate of 34% (Figure 5b). These results suggest that *ZmSNAC06*-T02 might regulate the plant adaptation to drought stress in maize.

Under drought stress, reactive oxygen species (ROS) are accumulated that could damage plant cells. Antioxidant enzymes are involved in the ROS scavenging system and protect plants from damage. Therefore, the three physiological indexes of the proline (Pro) content, superoxide dismutase (SOD) activity and malondialdehyde (MDA) content were measured in the WT and transgenic *Arabidopsis* before and after 10 days of drought treatment (Figure 5c–e). After drought treatment, the content of Pro in the three overexpression lines reached 304.29 mg/mg, 346.41 mg/mg and 333.67 mg/mg, respectively, all of which were significantly higher than the 240.50 mg/mg measured in the WT. Similarly, under drought stress, the SOD activity of transgenic *Arabidopsis* was higher than that of the WT. Then, we found that compared with the WT, the MDA content in transgenic *Arabidopsis* was significantly lower.

## 3. Discussion

### 3.1. Multitranscript Characteristics in ZmSNAC06

Multiple transcripts from a gene are usually the result of alternative splicing, which is widely involved in a variety of metabolic pathways within the plant genome [40]. Sequence analysis showed that *ZmSNAC06* has a special structure characterized by five transcripts, which may be the result of the coexistence of two splicing mechanisms, the intron and exon definition models. When long exons are separated by short (<250 bp) introns, such as in the *ZmSNAC06*-T01 transcript, pairing between splice sites takes place across an intron, and this process is called the intron definition model. On the other hand, in the *ZmSNAC06*-T02 transcript, with a long second intron of up to 604 bp, the splicing machinery is more likely to form across an exon than across an intron [41].

It was speculated that the multiple transcripts of *ZmSNAC06* were not caused by alternative splicing, but by alternative translation initiation. The mature mRNA of eukaryotes enters the cytoplasm for translation after being transcribed in the nucleus, so alternative translation initiation is the result of two splicing mechanisms acting on the same pre-mRNA. The qRT-PCR results showed that multiple transcripts of *ZmSNAC06* showed a synergistic response to drought stress in drought-tolerant and -sensitive materials, but their responses varied among different materials.

There are reports that multiple transcripts of genes perform different functions driven by different factors. For example, *FLM* (*Flowering Locus M*) has two subtypes of transcripts. Under high-temperature conditions, the expression of FLM-β decreased while the expression of FLM-α increased, thus affecting plant flowering [42]. It is worth noting that different transcripts of *ZmSNAC06* can be driven by drought and play drought-tolerant functions. It is speculated that the original transcript of *ZmSNAC06* prior to gene differentiation might have had a strong drought-tolerant function, leading to the preservation of a conserved drought-tolerant function in multiple differentiated transcripts.

### 3.2. ZmSNAC06-T02 Is an NAC Transcription Factor That Regulates Drought Tolerance

The structure of a transcription factor usually consists of four functional regions: a DNA-binding region, a transcriptional regulatory region, a nuclear localization signaling region and an oligomerization site region for the interaction of a transcription factor with other transcription factors or proteins [43]. *ZmSNAC06*-T02 contains a typical NAC conserved domain in the N-terminal region, and five subdomains have been identified in this domain. ZmSNAC06-P02 has a complete NAC domain, resulting in its localization in the nucleus. ZmSNAC06-P01 has only subdomain E and lacks a nuclear localization signal region, but a transactivation assay showed that the consistency of the C-terminal region of ZmSNAC06-P01/02 ensured that both had transactivation activity without being affected by different NAC domains.

Some members of the NAC transcription factor family have been reported to be involved in many plant regulation and development processes, including the regulation of drought stress. In maize, the drought tolerance function of *ZmNAC55*, *ZmNAC33*, *ZmNAC111*, *ZmNAC48*, *ZmNAC84*, *ZmNAC4*, *ZmNAC19*, *ZmNAC87*, *ZmJUB1*, *ZmNAP* and *ZmSNAC1* has been verified in transgenic plants [27,28,31,44,45,46,47]. Phylogenetic analysis showed that *ZmSNAC06*-T02 was more closely related to the known stress-responsive gene *OsNAC5*. The overexpression of *OsNAC5* increased tolerance to drought in rice at the vegetative stage, and more importantly, the root-specific rather than whole-body expression of *OsNAC5* increased the grain yield under drought conditions [48,49]. The analysis of the *ZmSNAC06*-T02 promoter sequence also showed that there were several binding sites of TFs related to a stress response in the promoter. The overexpression of *ZmSNAC06*-T02 made transgenic *Arabidopsis* more sensitive to ABA than the WT, suggesting that it may be involved in ABA-dependent signaling pathways in response to drought stress. It was also verified in our study that compared with the WT, transgenic *Arabidopsis* showed stronger drought tolerance and higher survival rates.

On the other hand, ROS are essential for plant growth and development. During normal physiological metabolism, the ROS system including production and removal in plants is in a state of equilibrium. However, drought stress can lead to the accumulation of excess ROS, which results in damage to plant cells [50]. Antioxidant enzymes such as MDA, SOD and Pro are known to play vital roles in ROS scavenging. After drought treatment, we also found that compared with the WT, the content of Pro increased, the activity of SOD increased and the content of MDA decreased in overexpression lines. This indicated that the *ZmSNAC06*-T02 transgenic lines had the ability to scavenge reactive oxygen species and suffered less damage under drought stress. In summary, the bioinformatics analysis, combined with the experimental results, indicates that *ZmSNAC06*-T02 is an NAC transcription factor responding to drought stress.

## 4. Materials and Methods

### 4.1. Plant Materials and Stress Treatment

The drought-tolerant inbred maize lines Tie7922 and X178, drought-sensitive inbred lines Ji81162 and CA339 and the evaluation of drought tolerance were provided by the Institute of Crop Sciences, Chinese Academy of Agricultural Sciences [51]. They were subjected to drought-stressed conditions at the V10 stage, early-developing young tassels were taken under well-watered and drought-stressed conditions at the V13 stage and total RNA samples were sequenced at the Beijing Genomics Institute, Shenzhen, China. Libraries were sequenced on the Illumina HiSeq 2000 Platform (Illumina, San Diego, CA, USA) according to the manufacturer’s recommendations [52].

Seeds of the Tie7922 and Ji81162 inbred lines, which had been surface-sterilized and germinated, were rolled in filter paper and placed vertically in distilled water in darkness for 3 days and then cultivated with Hoagland solution under a 16 h light/8 h dark cycle at 26 °C. At the three-leaf stage, the seedlings were transferred into Hoagland solution containing 20% (*w*/*v*) PEG-6000 for the drought treatment, and samples were collected after 0, 1, 3, 6 and 12 h of treatment. All samples were immediately frozen in liquid nitrogen and stored at −80 °C for RNA extraction.

### 4.2. Transcriptome Analysis

Transcriptome data of the Tie7922, X178, Ji81162 and CA339 inbred lines under well-watered and drought-stressed conditions were further analyzed. Digital gene expression counts were normalized through log_2_ transformation and compared using the reads per kilobase transcriptome per million mapped reads method (RPKM). A false discovery rate (FDR) ≤ 0.001, an absolute value of the Log_2_ Ratio ≥ 1 and *p* ≤ 0.001 were used as the thresholds to assess the significance of differences in gene expression. The KEGG (Kyoto Encyclopedia of Genes and Genomes) enrichment analysis was conducted using TBtools to compare the differences in the response pathways of the four materials under drought-stressed conditions (https://bioinfogp.cnb.csic.es/tools/venny/index.html, accessed on 29 October 2024). The GO (Gene Ontology) enrichment and gene family analyses of up-regulated response genes in drought-tolerant materials were performed using TBtools (https://dycharts.com/appv2/#/pages/home/chart-template, accessed on 29 October 2024).

### 4.3. Bioinformatics Analysis of ZmSNAC06

The sequences of *ZmSNAC06* and the NAC members of maize and other species were downloaded from the database website (https://maizegdb.org/, accessed on 29 October 2024; https://ensembl.gramene.org/Zea_mays/Info/Index, accessed on 29 October 2024). Multiple alignments of amino acid sequences were constructed with MEGA 11.0 software using ClustalW; the phylogenetic tree was constructed with MEGA 11.0 software using the neighbor-joining (NJ) method. Bootstrapping was carried out to obtain 1000 replicates with the pairwise deletion option. The PlantPAN4.0 website (http://plantpan.itps.ncku.edu.tw/plantpan4/index.html, accessed on 29 October 2024) was used to predict the binding sites of transcription factors within 2000 bp of the sequence upstream from the translation initiation codon (ATG) of *ZmSNAC06*.

### 4.4. RNA Extraction and Quantitative Real-Time PCR

Total RNA was extracted using the TransZol UP reagent. The total RNA was reverse-transcribed into cDNA with a qualified quality and concentration using the FastQuant RT Kit (Tiangen, Beijing, China). Quantitative real-time PCR (qRT-PCR) was performed using Applied Biosystems 7500 (Waltham, MA, USA), and the specificity of each primer pair was verified through melting curve analysis. TUB4/Actin was selected as an internal control, and gene expression was calculated using the 2^−ΔΔCt^ method with the variation in expression being estimated from three biological replicates. The primer pairs used for qRT-PCR analysis are listed in Supplementary Table S1.

### 4.5. Subcellular Localization and Transactivation Assay in Yeast Cells

The coding region of different transcripts of *ZmSNAC06* without the stop codon (TGA) was amplified using pairs of primers (Supplementary Table S1) and inserted into PAN580 vectors digested with *BamH*I to generate the ZmSNAC06-GFP fusion protein, respectively. The protoplast extraction and transformation methods were used with reference to the scheme of *Arabidopsis* [53]. The PAN580 vector was used as a negative control. The fluorescence of the GFP was observed through confocal microscopy after 12-16 h of incubation in darkness at room temperature.

The coding region of different transcripts of *ZmSNAC06* was amplified and inserted into pGBKT7 vectors digested with *EcoR*I and *BamH*I, respectively (the primers are listed in Supplementary Table S1). The fusion plasmid and the empty vector pGADT7-T were co-transformed into Y2HGlod yeast cells according to the manufacturer’s protocol. The empty vectors pGBKT7 and pGADT7-T were co-transformed as a negative control. The transformants were incubated on an SD/-Trp/-Leu or SD/-Trp/-Leu/-His/-Ade/X-α-Gal medium at 30 °C for 48–96 h.

### 4.6. Transformation of Arabidopsis and Drought Tolerance in Transgenic Arabidopsis

The coding region of *ZmSNAC06*-T02 was amplified from the inbred maize line Tie7922 through PCR (the primers are listed in Supplementary Table S1). The *Arabidopsis* ecotype Col-0 was transformed using the floral dipping method. For the selection of transformants, T_0_ seeds were plated on a 1/2 Murashige and Skoog (MS) medium with 1% sucrose and 50 mg/mL hygromycin B. Homozygous T_3_ plants were used for further analysis.

Wild-type (WT) and *ZmNAC06*-overexpressed transgenic seedlings that germinated on 1/2 MS medium for one week were transferred into pots containing a soil mixture (vermiculite/nutrient soil = 3:1). They were grown under normal conditions for 2 weeks in a growth chamber under a 16 h light/8 h dark cycle at 22 °C, then water was withheld from the plants for 2 weeks. Watering was then resumed to allow the plants to recover. Three days later, the number of surviving plants was recorded. At least 16 plants of each line were compared with the WT in each test, and statistical data were based on data obtained from three independent experiments.

### 4.7. Germination and Determination of Physiological Indexes Under Stress

One hundred seeds of WT and *ZmSNAC06*-overexpressed plants were sown on a 1/2 MS medium with or without 1 μM ABA, respectively. There were three replicated experiments. Plates were chilled at 4 °C for 3 days to synchronize germination and moved to 22 °C with a 16 h light/8 h dark cycle. Germination was scored on the seventh day after germination.

To detect changes in physiological indices, leaves were collected from plants before and after 10 days of drought treatment. The contents of MDA and Pro and the activity of SOD in the leaves of *Arabidopsis* were measured with the corresponding detection kits (Solarbio, Beijing, China).

### 4.8. Statistical Analyses

Statistical analysis was performed using Microsoft Excel. All values are the mean (±SD) of three biological replicates. Significant differences were determined by a *t*-test (* *p* < 0.05, ** *p* < 0.01).

## 5. Conclusions

In this study, we identified the *ZmSNAC06* gene between drought-tolerant (Tie7922 and X178) and drought-sensitive (Ji81162 and CA339) inbred lines through RNA-seq analysis. Bioinformatic analysis revealed that *ZmSNAC06*, which had five transcripts, belonged to the NAC transcription factor family. *ZmSNAC06*-T02 had a typical NAC domain. ZmSNAC06-P02 was localized in the nucleus of maize protoplasts and had transactivation activity in yeasts. The overexpression of *ZmSNAC06*-T02 in *Arabidopsis* resulted in hypersensitivity to ABA during germination, increased antioxidant enzyme activities to keep the balance of ROS and enhanced drought tolerance in transgenic *Arabidopsis*. These findings help us understand the function of *ZmSNAC06* and provide a theoretical basis for cultivating drought-tolerant varieties in the future.

## Data Availability

All of the datasets are included within the article and its additional files.

## Supplementary Materials

The following supporting information can be downloaded at https://www.mdpi.com/article/10.3390/plants14010012/s1: Supplementary Table S1: Primer sequence; Supplementary Figure S1: Differences in the ZmSNAC06 sequence between the two inbred lines. (a) Differences in sequence in gene region. The red region is the exon region, and the gray area is the intron region. (b) Differences in amino acid sequence.
