# Supplementary material for: Identification of ZmSNAC06, a Maize NAC Family Transcription Factor with Multiple Transcripts Conferring Drought Tolerance in Arabidopsis
Source: Plants (Basel). 2024 Dec 24;14(1):12. doi: 10.3390/plants14010012 (PMC11722792; doi:10.3390/plants14010012)
Supplement: Supplementary file 1 [file plants-14-00012-s001.zip › plants-3312815-supplementary.pdf]

Supplementary Table S1. Primer sequence

Primer name	Forward primer	Reverse primer
ZmSNAC06-T01-UTR	CTTGGGCAGGATAGGCTC	GCACGGTGGTGTTGGTTT
ZmSNAC06-T01-ORF	ATGGCCGGCGCAGAG	TCAGAACGGTTTGTGCAGGT
ZmSNAC06-T02-UTR	TCAGCAATCAATCAGCTTTCC	GTTGGTTTCGCAGCACTACAC
ZmSNAC06-T02-ORF	ATGGACTGCGGTGGCGC	TCAGAACGGTTTGTGCAGG
ZmSNAC06-CDS	CCGGGTACAGCTGCGCGATGT<br>T	GAGCACTCCTGGTTTCTGAAA<br>G
Promoter-wy	CCACCTCAATCCACTCCA	CACTCCTTCTCCCCACCG
Promoter-2000	ACACCCTCCAGCCGCGT	GGAACATCGCGCAGCTGTAC
ZmSNAC06-OE	<u>GGTAGATCTGACTAGT</u><br>ATGGACTGCGGTGGCGC	<u>CAGGACGTAAACTAGT</u><br>GAACGGTTTGTGCAGGTAC
Pan580-T01	<u>GGACCGGTCCCGGGGGATCC</u><br>ATGGCCGGCGCAGAG	<u>CTCGCCCTTGCTCACCAT</u><br>GAACGGTTTGTGCAGGTACG
Pan580-T02	<u>GGACCGGTCCCGGGGGATCC</u><br>ATGGACTGCGGTGGCGC	<u>CTCGCCCTTGCTCACCAT</u><br>GAACGGTTTGTGCAGGTAC
BD-T01	<u>ATGGAGGCCGAATTC</u><br>ATGGCCGGCGCAGAG	<u>CAGGTCGACGGATCC</u><br>GAACGGTTTGTGCAGGTACG
BD-T02	<u>ATGGAGGCCGAATTC</u><br>ATGGACTGCGGTGGCGC	<u>CAGGTCGACGGATCC</u><br>GAACGGTTTGTGCAGGTAC
ZmSNAC06-qrt-T01	GACCTCATCTCCAAGCCTCT	TCGTACCGCTCGATAACACC
ZmSNAC06-qrt-T02	AGTGGTACTTCTTCTCGCCGC	TCGTGCATGATCCAGTTGGTC
ZmSNAC06-Jd	TGGAGAGAACACGGGGGACT	CGGCGAACTGATCGTTAAAA

Note: The underlined part is the homologous arm sequence of the vector.

a

	58	98-103	188	719	740	767	796	859	1198	1214	1227	1228	1230	1259	1329	1338	1363	1730-1741	1808-1813	1851	1938-1949	2034	2102	2113	2135-2143	2174-2175	2178	2365	2631
Tie7922	C	CCGCCT	T	G	-	G	A	G	T	-	A	G	C	A	C	T	G	CCGCCGCCGCCG	TCGACG	G	--CGCGA-----	A	C	-	---------	GT	A	-	G
Ji81162	G	------	C	G	C	A	-	G	C	A	C	T	T	T	T	C	A	CCGCCG------	------	T	--CGCGACGCGA	T	T	A	GGCTGCGC	--	G	T	T

b

```
Tie7922    MDCGGALQLPPGFRFHPTDDELVMYYLLRKCGGLPLAAPVIAEVDLYKFDPWQLPEKAFGGEKEW    65
Ji81162    MDCGGALQLPPGFRFHPTDDELVMYYLLRKCGGLPLAAPVIAEVDLYKFDPWQLPEKAFGGEKEW    65
Consensus  mdcggalqlppgfrfhptddelvmyyllrkcgglplaapviaevdlykfdpwqlpekafggekew

Tie7922    YFFSPRDRKYPNGSRPNRAAGTGYWKATGADKPVGSPRPVAIKKALVFYAGKPPKGVKTNWIMHE    130
Ji81162    YFFSPRDRKYPNGSRPNRAAGTGYWKATGADKPVGSPRPVAIKKALVFYAGKPPKGVKTNWIMHE    130
Consensus  yffsprdrkypngsrpnraagtgywkatgadkpvgsprpvaikkalvfyagkppkgvktnwimhe

Tie7922    YRLADVDRSAAARKKTNNALRLDDWVLCRIYNKKGVIERYDTVDDDDGDGAVAEDVKPVPAPAAS    195
Ji81162    YRLADVDRSAAARKKTNNALRLDDWVLCRIYNKKGVIERYDTVNDDDGDGAVAEDVKPVPAPAAS    195
Consensus  yrladvdrsaaarkktnnalrlddwvlcriynkkgvierydtv dddgdgavaedvkpvpapaas

Tie7922    KNPRASARVGAAAAAPMKVELPEYGGGYYDYDDLETPSAGMLCFDRPSAPAPADRDSNNSVPWTT    260
Ji81162    KNPRASARVGAAAAAPMKVELPEYGGGYYDYDDLETPSAGMLCFDRPSAPAPADRDSNNSVPWTT    260
Consensus  knprasarvgaaaaapmkvelpeygggyydyddletpsagmlcfdrpsapapadrdsnnsvpwtt

Tie7922    HHTHTDNSSSGSERVLSPSPDLPDRDHAESQPAAAAAAAGWWPVGGDWGSAAEDGFMVDVDVDDG    325
Ji81162    HHTHTDNSSSGSERVLSPSPDLPDRDHAESQP..AAAAAGWWPVGGDWGSAAEDGFM..VDVDDG    321
Consensus  hhthtdnsssgservlspspdlpdrdhaesqp  aaaaagwwpvggdwgsaaedgfm  vdvddg

Tie7922    SSLFGPLSPGLFARVDAAAFGFGDMLASYLHKP                                    358
Ji81162    SSLFGPLSPGLFARVDAAAFGFGDMLASYLHKP                                    354
Consensus  sslfgplspglfarvdaaafgfgdmlasylhkp
```

Supplementary Figure S1. Differences of *ZmSNAC06* sequence between the two inbred lines. (**a**) Differences of sequence in gene region. The red region is the exon region, and the gray area is the intron region. (**b**) Differences of amino acid sequence.
